# Risk of secondhand smoke exposure and severity of COVID-19 infection: multicenter case–control study

**DOI:** 10.3389/fpubh.2023.1210102

**Published:** 2023-08-03

**Authors:** Surekha Kishore, Vandana Shah, Om Prakash Bera, U. Venkatesh, Rakesh Kakkar, Pradeep Aggarwal, Pankaj Bhardwaj, C. M. Singh, Chetna Maliye, Suneela Garg, Geetha R. Menon, Puneet Misra, Shival Kishore Verma, The COVID SHS Study Group, D.K. Srivastava

**Affiliations:** ^1^All India Institute of Medical Sciences, Gorakhpur, Uttar Pradesh, India; ^2^Campaign for Tobacco-Free Kids, New Delhi, India; ^3^Global Health Advocacy Incubator, New Delhi, India; ^4^Department of Community Medicine and Family Medicine, All India Institute of Medical Sciences, Gorakhpur, Uttar Pradesh, India; ^5^Department of Community and Family Medicine, All India Institute of Medical Sciences, Bathinda, Punjab, India; ^6^Department of Community and Family Medicine, All India Institute of Medical Sciences, Rishikesh, Uttarakhand, India; ^7^Department of Community Medicine and Family Medicine, All India Institute of Medical Sciences, Jodhpur, Rajasthan, India; ^8^Department of Community and Family Medicine, All India Institute of Medical Sciences, Patna, Bihar, India; ^9^Department of Community Medicine, Mahatma Gandhi Institute of Medical Sciences, Sevagram, Maharashtra, India; ^10^Department of Community Medicine, Maulana Azad Medical College and Associated Hospitals, New Delhi, India; ^11^ICMR-National Institute of Medical Statistics, New Delhi, India; ^12^Centre for Community Medicine, All India Institute of Medical Sciences, New Delhi, India; ^13^Johns Hopkins Bloomberg School of Public Health, Baltimore, MD, United States

**Keywords:** secondhand smoke, COVID-19, smoking, vaccination, substance abuse

## Abstract

**Introduction:**

Exposure to secondhand smoke (SHS) is an established causal risk factor for cardiovascular disease (CVD) and chronic lung disease. Numerous studies have evaluated the role of tobacco in COVID-19 infection, severity, and mortality but missed the opportunity to assess the role of SHS. Therefore, this study was conducted to determine whether SHS is an independent risk factor for COVID-19 infection, severity, mortality, and other co-morbidities.

**Methodology:**

Multicentric case–control study was conducted across six states in India. Severe COVID-19 patients were chosen as our study cases, and mild and moderate COVID-19 as control were evaluated for exposure to SHS. The sample size was calculated using Epi-info version 7. A neighborhood-matching technique was utilized to address ecological variability and enhance comparability between cases and controls, considering age and sex as additional matching criteria. The binary logistic regression model was used to measure the association, and the results were presented using an adjusted odds ratio. The data were analyzed using SPSS version 24 (SPSS Inc., Chicago, IL, USA).

**Results:**

A total of 672 cases of severe COVID-19 and 681 controls of mild and moderate COVID-19 were recruited in this study. The adjusted odds ratio (AOR) for SHS exposure at home was 3.03 (CI 95%: 2.29–4.02) compared to mild/moderate COVID-19, while SHS exposure at the workplace had odds of 2.19 (CI 95%: 1.43–3.35). Other factors significantly related to the severity of COVID-19 were a history of COVID-19 vaccination before illness, body mass index (BMI), and attached kitchen at home.

**Discussion:**

The results of this study suggest that cumulative exposure to secondhand cigarette smoke is an independent risk factor for severe COVID-19 illness. More studies with the use of biomarkers and quantification of SHS exposure in the future are needed.

## 1. Introduction

Globally, more than 460 million SARS-CoV-2 infection (COVID-19) cases, with 6 million deaths, have been reported in people of all age groups (1). COVID-19 has been known to cause severe respiratory illness and multi-organ inflammatory disease (2). Smoking, cardiovascular disease (CVD), hypertension, and chronic lung diseases are risk factors for COVID-19 severity (3–5). Older individuals, as well as those of any age with co-morbidities, such as hypertension and diabetes, have exhibited a poorer prognosis (6). Individuals with chronic obstructive pulmonary disease (COPD) or other respiratory illnesses are at a greater risk for severe illness and have been associated with hospitalization and intensive care unit (ICU) admissions (7). Tobacco smoke comprises over 7,000 chemicals, including 69 compounds known to be carcinogenic and other harmful chemicals such as hydrogen cyanide, carbon monoxide, and ammonia (8). Tobacco use including smoking is associated with increased severity/progression of disease, mortality, and hospitalized COVID-19 patients (9–11). Secondhand smoke (SHS) is a combination of tobacco smoke exhaled by smokers during the smoking of cigarettes, bidi, hookah, etc., and smoke produced from the burning ends of these products. Thirdhand smoke is a residue from firsthand and SHS on clothes, furniture, and household surfaces (12). SHS is responsible for approximately 1.2 million deaths globally each year (13). Adults who are exposed to SHS have increased rates of coronary heart disease, stroke, and lung cancer; additionally, babies born to mothers who smoke are more likely to die of sudden infant death syndrome (SIDS), prematurity, low birth weight, and other conditions (14, 15). Moreover, it is a fact that smokers are less aware of the ill effects of SHS when compared to smokers (16). Exposure to SHS is an established causal risk factor for cardiovascular disease (CVD) and chronic lung disease (17) and may also be a risk factor for COVID-19 severity, either by contributing to the development of these underlying conditions or by triggering inflammation and upregulation of angiotensin-converting enzyme 2 (ACE-2) receptors, which facilitate the entry of COVID-19 into cells (5). However, a lack of SHS surveillance makes it difficult to quantify any potential relationship or to make evidence-informed recommendations about the effects of SHS exposure on COVID-19 incidence or severity (5). Numerous studies have evaluated the role of tobacco in COVID-19 infection, severity, and mortality but missed the opportunity to assess the role of SHS (18). Thus, this study has been planned to study SHS as an independent risk factor for COVID-19 infection, severity, mortality, and other co-morbidities. With this background, we conducted a case–control study to determine the association of COVID-19 severity with SHS exposure SHS and to evaluate the relationship between the clinical-social profile of COVID-19 patients and exposure to SHS (19).

## 2. Methods

### 2.1. Study design and sites

**Figure 1:**
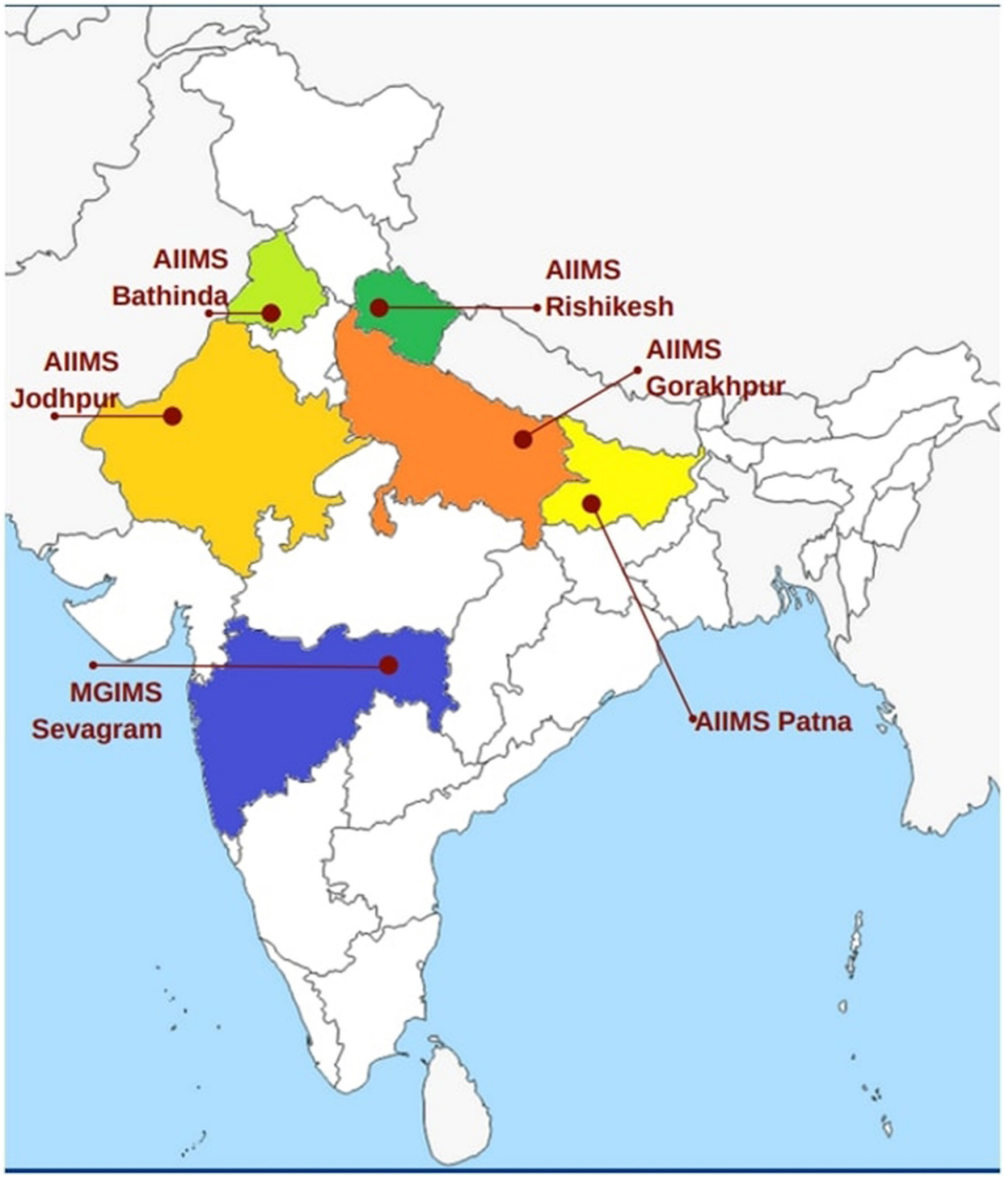
Sites that participated in the study.

After obtaining approval from the Institutional Ethics Committee, this case–control study was conducted retrospectively by choosing persons with COVID-19 admitted and non-admitted from multiple sites across India (Figure 1).

### 2.2. Study participants

All the cases were ≥ 18 years of age for both sexes and were from rural as well as urban areas. We evaluated the exposure to SHS in our study, comparing severe COVID-19 patients as cases and mild and moderate COVID-19 patients as controls. The recruited cases were laboratory-confirmed COVID-19 patients with severe manifestations between 1 January 2020 and 28 February 2022. The cases were contacted and traced from the inpatient records available in the study sites. The control group participants in this research study were frequently matched to the cases based on gender, age at the time of recruitment, and neighborhood-matched control selection was done with each case to minimize ecologic variability. Active smokers, individuals with incomplete COVID-19 diagnosis information, and those who did not provide consent were excluded from both the case and control groups.

### 2.3. Collection of epidemiological data

After obtaining written consent, trained interviewers conducted face-to-face interviews with all participants using a questionnaire that covered topics such as past and current exposure to SHS, demographic details, lifestyle habits (including alcohol consumption), and both occupational and home exposure to SHS. Participants self-reported exposure to SHS was assessed by asking whether anyone who had ever lived in their household was a smoker, either currently or in the past (such as parents, grandparents, siblings, or others), and if so, whether they regularly smoked in the home during their lifetime. Similar questions were asked about the regular smoking habits of others in the household during the participant's childhood. The participants were also asked to provide information about their age at the time of exposure to SHS and the number of active smokers in their households.

### 2.4. Measures and specification

- **Secondhand smoke:** SHS is the combination of smoke from the burning end of a cigarette, and the smoke breathed out by smokers (20).
- **COVID-infected cases:** A person with laboratory confirmation of COVID-19 infection (RT PCR Positive), irrespective of clinical signs and symptoms.
- **Severe COVID-19:** The severity of COVID-19 was defined as patients admitted with COVID-19 having SpO2 <90% on room air at sea level or COVID-19 Reporting and Data System (CO-RADS) on Chest Computed Tomography 4 or more at the time of admission. Patients with COVID-19 were considered to have severe illness if they have SpO2 <94% on room air at sea level, PaO2/FiO2 <300 mm Hg, a respiratory rate >30 breaths/min, or lung infiltrates >50%. These patients may experience rapid clinical deterioration (21, 22).
- **Mild/Moderate COVID-**19: Patients who were diagnosed with COVID-19 but have not shown signs of severe COVID-19.
- **Smoking:** Current Smoker user is an adult who has smoked 100 cigarettes in his or her lifetime and currently smokes cigarettes (until the COVID-19 illness). A former smoker user was an adult who had smoked at least 100 cigarettes in his or her lifetime but who had quit smoking at the time of the interview. Never smoker was an adult who has never smoked, or who has smoked less than 100 cigarettes in his or her lifetime (23).
- **SHS exposure at Home:** Exposure at home was estimated for non-smokers who reported anyone smoking inside his/her home (that excludes areas outside, such as patios, balconies, gardens, etc., that are not fully enclosed). SHS was taken to be present (“Yes”) if the response to the question “*How often does anyone smoke inside your home*?” was “more than or equal to once in the last 1 month” (24).
- **SHS exposure at the workplace:** This was assessed for the respondents who work outside of the home and who usually work indoors or both indoors and outdoors. SHS is defined as the percentage of respondents who reported someone smoking at least in indoor workplaces in the past 30 days before the survey. SHS was considered to be present (“Yes”) if the response to the question “During the past 30 days- did anyone smoke in indoor areas where you work?” was affirmative (24).

### 2.5. Statistical analysis

Statistical analysis was performed using IBM SPSS Statistics Software (version 26; IBM, New York, USA). Quantitative data were presented as mean with standard deviation (SD) and counting data were presented as the percentage of the total unless otherwise specified. The comparisons of the quantitative data were statistically evaluated using the parametric or non-parametric test, according to the distribution which was assessed by the Shapiro–Wilk test.

We used independent *t*-tests to measure the differences between the two groups (cases vs. controls) for continuous variables and Pearson's chi-square to test the correlations between the study groups for categorical parameters. We used binary logistic regression models to test correlations between the study groups and SHS exposure variables with adjustments for significant and potential confounders. The *p*-value of 5% or less was considered statistically significant. The data were analyzed using SPSS version 24 (SPSS Inc., Chicago, IL, USA).

### 2.6. Sample size

The sample size was calculated using Epi-info version 7. Three Fleiss with continuity correction formula calculated the required sample size with a two-sided significance level (1-alpha) 95% and 0.80% Power (1-beta, % chance of detecting). With a controls/cases ratio of 1 and an odds ratio of 1.3, a total of 1,132 individuals are needed for the study, comprising 566 individuals in both the cases and control. The required sample size calculated using Fleiss or Fleiss with continuity correction formula was 1,070 and 1,132 persons, respectively. A minimum sample size of 1,250 individuals was determined for the study.

### 2.7. Research ethics

This research adheres to rigorous ethical standards, including obtaining approval from the Institutional Human Ethics Committee (IHEC) with the number IHEC/AIIMS-GKP/BMR/105/2022 at AIIMS Gorakhpur. Confidentiality, informed consent, participant wellbeing, and data protection were prioritized throughout the study.

## 3. Results

A total of 672 cases of severe COVID-19 and 681 controls of mild and moderate COVID-19 were recruited in this study. The socio-demographic data and other characteristics among cases and controls were compared using the chi-square test, as shown in Table 1. Participants who were above 30 years of age were significantly associated with severe COVID-19 compared to mild/moderate COVID-19, i.e., 81.4% (547/672) vs. 69.3% (472/672) (*P* < 0.001). Similarly, among other demographic variables, married individuals and illiterate were significantly associated with severe COVID-19 illness with a p-value of <0.001 and 0.02, respectively. Among the clinical characteristics, those with BMI ≥23.0, RBS ≥200, systolic blood pressure ≥140, and diastolic blood pressure ≥90 at the time of the interview were found to be significantly associated with severe COVID-19 illness. Also, it was found that among the severe cases, the history of vaccination was less frequent compared to the controls, i.e., 7.5% (42/563) vs. 21.5% (123/571) (*P* < 0.001).

**Table 1:** Socio-demographic and other characteristics of cases and controls.

**Characteristics**	**Case *N* (%)**	**Control *N* (%)**	*p*-value^*^
**Gender**			
Male	448 (66.7)	434 (63.7)	0.25
Female	224 (33.3)	247 (36.3)	
**Age**			
< 30 Yrs.	125 (18.6)	209 (30.7)	**< 0.01**
30 and above	547 (81.4)	472 (69.3)	
**Residence**			
Rural	335 (49.9)	347 (51.0)	0.68
Urban	337 (50.1)	334 (49.0)	
**Marital status**			
Married	592 (88.1)	523 (76.8)	**< 0.01**
Unmarried	74 (11.0)	154 (22.6)	
Widowed/divorced/separated	6 (0.9)	4 (0.6)	
**Education level**			
Illiterate	50 (7.4)	31 (4.6)	**0.02**
Literate	622 (92.6)	650 (95.4)	
**Socio-economic class**			
I (Upper)	390 (58.0)	415 (60.9)	0.05
II and III (upper middle and middle)	57 (8.5)	75 (11.0)	
IV and V (lower middle and lower)	225 (33.5)	191 (28.0)	
**COVID vaccination before COVID**			
Yes	42 (7.5)	123 (21.5)	**< 0.01**
No	521 (92.5)	448 (78.5)	
**No of doses of COVID vaccination before COVID**			
One dose	41 (7.3)	79 (13.8)	**< 0.01**
Both doses	1 (0.2)	44 (7.7)	
None	521 (92.5)	448 (78.5)	
**Body mass index (BMI)**			
< 23	141 (21.5)	231 (35.1)	**< 0.01**
≥23	515 (78.5)	427 (64.9)	
**Random blood sugar (RBS)**			
200 and above	36 (5.4)	16 (2.3)	**< 0.01**
< 200	636 (94.6)	665 (97.7)	
**Systolic blood pressure**			
140 and above	184 (27.4)	110 (16.2)	**< 0.01**
< 140	488 (72.6)	571 (83.8)	
**Diastolic blood pressure**			
90 and above	206 (30.7)	167 (24.5)	**< 0.01**
< 90	466 (69.3)	514 (75.5)	

* Pearson's Chi-square test.

Bold values mean that the result was statistically significant.

It was also found that exposure to SHS was comparatively more among participants with severe COVID-19 illness (cases) when compared to participants with moderate and mild COVID-19 illness (controls), i.e., 51.2% (344/672) vs. 27.6% (147/681) (*P* < 0.001). Similarly, the participants with severe COVID-19 illness reported more frequent exposure to secondhand smoking at the workplace compared to participants with mild and moderate COVID-19 illness, i.e., 15.5% (111/672) vs. 9.0% (61/681) (*P* < 0.001), as shown in Table 2.

**Table 2:** Exposure to tobacco smoke and other substances in cases and controls.

**Characteristics**	**Case *N* (%)**	**Control *N* (%)**	*p*-value^*^
**Is anyone in your house a smoker (parents, grandparents, siblings, etc.)?**			
Yes	344 (51.2%)	188 (27.6%)	**< 0.01**
No	328 (48.8%)	493 (72.4%)	
**Is anyone in your workplace who is a smoker?**			
Yes	111 (16.5%)	61 (9.0%)	**< 0.01**
No	561 (83.5%)	620 (91.0%)	
**Alcohol consumption (If 3 times/week or more: yes)**			
Yes	5 (0.7%)	5 (0.7%)	0.98
No	667 (99.3%)	676 (99.3%)	
**Kitchen**			
Attached	488 (72.6%)	524 (76.9%)	0.06
Separated	184 (27.4%)	157 (23.1%)	
**Kitchen chimney**			
Present	129 (19.2%)	154 (22.6%)	0.12
Absent	543 (80.8%)	527 (77.4%)	
**Cooking fuel**			
Gas	649 (96.6%)	649 (95.3%)	0.23
Wood	23 (3.4%)	32 (4.7%)	
**Is the house overcrowded?**			
Present	42 (6.3%)	36 (5.3%)	0.44
Absent	630 (93.8%)	645 (94.7%)	
**House adequately ventilated**			
Proper	638 (94.9%)	645 (94.7%)	0.85
Improper	34 (5.1%)	36 (5.3%)	

* Pearson's Chi-square test.

Bold values mean that the result was statistically significant.

To control all potential confounders, logistic regression analysis was performed, and it was observed that the adjusted odds ratio (AOR) for SHS exposure at home was 3.03 (CI 95%: 2.29–4.02) compared to mild/moderate COVID-19, while SHS exposure at the workplace had odds of 2.19 (CI 95%: 1.43–3.35) compare to control, i.e., mild/moderate illness. The COVID vaccination and BMI <23 were shown to be protective with an odds ratio of 0.24 (0.15–0.36) and 0.51 (0.38–0.71), respectively. Furthermore, other variables, such as systolic blood pressure and history of COVID-19 vaccination, also had a significant association with severe COVID-19 illness when compared to mild and moderate illness (*p*-value of < 0.01) (Table 3).

**Table 3:** Association of SHS exposure and other characteristics with case and control using logistic regression analysis.

**Characteristics**	**Case *N* (%)**	**Control *N* (%)**	**Unadjusted odd ratio**	Adjusted odds ratio^*^(AOR)	*p*-value^*^
**Age**					
< 30 Yrs.	125 (18.6)	209 (30.7)	0.52	1.06 (0.70–1.60)	0.76
30 and above	547 (81.4)	472 (69.3)	Ref.		
**Marital status**					
Married	592 (88.1)	523 (76.8)	**0.76**	0.66 (0.17–2.49)	0.54
Unmarried	74 (11.0)	154 (22.6)	**0.32**	0.27 (0.06–1.10)	0.06
Widowed/divorced/separated	6 (0.9)	4 (0.6)	Ref.		
**Sex**					
Male	448 (66.7)	434 (63.7)	1.138	1.17 (0.83–1.66)	0.34
Female	224 (33.3)	247 (36.3)	Ref.		
**Education level**					
Illiterate	50 (7.4)	31 (4.6)	1.69	1.30 (0.71–2.35)	0.38
Literate	622 (92.6)	650 (4.7)	Ref.		
**Socio–economic class**					
I (Upper)	390 (58.0)	415 (60.9)	0.8	0.58 (0.40–0.84)	0.04
II and III (upper middle and middle)	57 (8.5)	75 (11.0)	0.65	0.52 (0.31–0.87)	0.01
IV and V (lower middle and lower)	225 (33.5)	191 (28.0)	Ref.		
**Systolic blood pressure**					
140 and above	184 (27.4)	110 (16.2)	1.96	2.06 (1.38–3.06)	**< 0.01**
< 140	488 (72.6)	571 (83.8)	Ref.		
**Diastolic blood pressure**					
90 and above	206 (30.7)	167 (24.5)	1.36	0.86 (0.60–1.22)	0.41
< 90	466 (69.3)	514 (75.5)	Ref.		
**COVID vaccination before COVID**					
Yes	42 (7.5)	123 (21.5)	**0.29**	0.24 (0.15–0.36)	**< 0.01**
No	521 (92.5)	448 (78.5)	Ref.		
**Random blood sugar (RBS)**					
200 and above	36 (5.4)	16 (2.3)	0.86	3.29 (1.30–8.27)	0.011
< 200	636 (94.6)	665 (97.7)	Ref.		
**House adequately ventilated**					
Proper	638 (94.9%)	645 (94.7%)	2.35	0.40 (0.20–0.80)	0.010
Improper	34 (5.1%)	36 (5.3%)	Ref.		
**Is the house overcrowded?**					
Present	42 (6.3%)	36 (5.3%)	1.19	1.19 (0.65–2.18)	0.56
Absent	630 (93.8%)	645 (94.7%)	Ref.		
**Cooking fuel**					
Gas	649 (96.6%)	649 (95.3%)	1.39	1.17 (0.58–2.37)	0.64
Wood	23 (3.4%)	32 (4.7%)	Ref.		
**Kitchen chimney**					
Present	129 (19.2%)	154 (22.6%)	0.81	0.958 (0.68–1.33)	0.80
Absent	543 (80.8%)	527 (77.4%)	Ref.		
**Body mass index (BMI)**					
< 23	141 (21.5)	231 (35.1)	0.51	0.51 (0.38–0.71)	**< 0.01**
≥ 23	515 (78.5)	427 (64.9)	Ref.		
**SHS exposure at home**					
Yes	344 (51.2%)	188 (27.6%)	2.75	3.03 (2.29–4.02)	**< 0.01**
No	328 (48.8%)	493 (72.4%)	Ref.		
**SHS exposure at the workplace**					
Yes	111 (16.5%)	61 (9.0%)	2.01	2.19 (1.43–3.35)	**< 0.01**
No	561 (83.5%)	620 (91.0%)	Ref.		
**Alcohol consumption (If 3 times/week or more: yes)**					
Yes	5 (0.7%)	5 (0.7%)	1.01	1.02 (0.21–5.00)	0.97
No	667 (99.3%)	676 (99.3%)	Ref.		
**Do you currently use/ever used tobacco?**					
Yes	9 (1.3%)	8 (1.2%)	1.14	0.38 (0.10–1.38)	0.14
No	663 (98.7%)	673 (98.8%)	Ref.		
**Residence**					
Rural	335 (49.9)	347 (51.0)	0.96	0.93 (0.70–1.23)	0.63
Urban	337 (50.1)	334 (49.0)	Ref.		

* Model was adjusted for age (below and above 30 years of age), sex (male, female), education level (literate or illiterate), socio-economic class, systolic blood pressure (≥140 mm of hg), diastolic blood pressure (≥90 mm of hg), status of COVID vaccination, random blood sugar (RBS ≥200), adequate ventilation of home, presence of kitchen chimney, BMI (≥23), SHS exposure at home, SHS exposure at workplace, alcohol consumption (Yes/No), tobacco use (Yes/No), and residence (Urban/Rural).

Bold values mean that the result was statistically significant.

## 4. Discussion

In this case–control study, after controlling all confounders related to socio-demographic and clinic-social characteristics, we found that those who had severe COVID-19 illness had 3.03 times greater odds of being exposed to SHS at their home when compared to controls. Also, the exposure to SHS at the workplace was 2.19 times greater among participants with severe COVID-19 illness compared to participants with mild and moderate COVID-19 illness. Furthermore, it was also noticed that participants who were severely ill with COVID-19, when compared to participants with mild and moderate illness due to the same were less likely to have a previous history of COVID-19 vaccination.

To the best of our knowledge, this study is the first to associate secondhand exposure with the severity of COVID. Our study demonstrated secondhand smoking as an independent risk factor for severe COVID illness. Our research results were also consistent with other studies in Asian countries where the level of tobacco exposure at home and in the environment was high (8–13). Also, as mentioned in WHO's Global estimate of the burden of disease from SHS (25), 9% of the global lower respiratory tract infection risk could be attributed to SHS. In addition, our study's findings somewhat align with those of a study performed by Jasper V. Been et al. (26), on exposure to SHS and reduction in hospitalization due to lower respiratory tract infection. Similarly, a study by Flouris et al. (27) also claims that even 1-h exposure to SHS produces deleterious effects on our lung membrane, resulting in exaggerated respiratory tract infection symptoms. Other factors that were found to be associated with severe COVID-19 illness are BMI >23 kg/m^2^ and systolic blood pressure of 140 mm of hg or above. Some previous literature also suggests some other factors that are independently related/associated with the severity of COVID-19 illness such as obesity, low education level, and diet. Albashir AAD (28) mentioned in his study that the most common characteristics of those hospitalized due to severe COVID-19 illness were diabetes, chronic lung disease and cardiovascular disease, and obesity is a well-known risk factor for these diseases. One of the key points of his study was that obesity is a strong independent risk factor for hospitalization in COVID-19 and increases the need for critical care and invasive mechanism (28, 29). It is also found in other studies, in addition to factors such as diabetes, diet, and nutrient intake, such as supplementation with omega-3 fatty acids and fish consumption (30, 31).

## 5. Strength and limitations

The strengths of this study were that we recorded and controlled environmental factors, vaccination status, and clinical characteristics in our analysis, thus eliminating the major confounders. Neighborhood matching was made for the selection of controls thus minimizing ecological bias along with age and sex.

The limitations of this study were as follows: First, SHS exposure was estimated through face-to-face interviews; it was neither possible to measure nicotine levels in hair nor to measure cotinine in urine to determine tobacco exposure. Second, as this study was conducted during the COVID-19 lockdown, SHS exposure at social events could not be recorded. Third, due to the retrospective nature of case–control studies, the questionnaires were administered after the COVID-19 illness occurred, and therefore bias cannot be excluded. Finally, we cannot collect all possible confounders for the severity of COVID-19, as it is a memory bias. More studies using biomarkers and quantifying SHS exposure in the future are needed to confirm this finding. The results of this study suggest that cumulative exposure to secondhand cigarette smoke is an independent risk factor for hospital admission from COVID-19.

## Data availability statement

The raw data supporting the conclusions of this article will be made available by the authors, without undue reservation.

## Ethics statement

The studies involving human participants were reviewed and approved by Institutional Ethical Committee, AIIMS Gorakhpur Similar ethical permission was obtained from all the other sites before the commencement of data collection. The patients/participants provided their written informed consent to participate in this study. Written informed consent was obtained from the individual(s) for the publication of any potentially identifiable images or data included in this article.

## Author contributions

Conceptualization: SKi, VS, OB, and UV. Data curation and investigation: SKi, UV, RK, PA, PB, CS, and CM. Formal analysis: UV, OB, and GM. Supervision: SKi, VS, OB, UV, CM, SG, GM, and PM. Validation: SKi, UV, RK, PA, PB, CS, CM, GM, PM, and SG. Writing—original draft: UV, VS, OB, RK, PA, and PB. Writing—review and editing: SKi, UV, CS, CM, SG, GM, and PM. All authors contributed to the article and approved the submitted version.

## COVID SHS study group

Dr. D.K. Srivastava (Professor and HoD, Department of Community Medicine, Baba Raghav Das Medical College Gorakhpur, Uttar Pradesh, India), Dr. Anuj Mundra (Department of Community Medicine, Mahatma Gandhi Institute of Medical Sciences, Sevagram, Maharashtra, India), Dr. Amey Dhatrak (Department of Community Medicine, Mahatma Gandhi Institute of Medical Sciences, Sevagram, Maharashtra, India), Dr. Mahendra Singh Gehlot (Department of Community and Family Medicine, All India Institute of Medical Sciences, Rishikesh, Uttrakhand, India), Dr. Yogesh Bahurupi (Department of Community and Family Medicine, All India Institute of Medical Sciences, Rishikesh, Uttrakhand, India), Dr. Santosh Kumar Nirala (Department of Community and Family Medicine, All India Institute of Medical Sciences, Patna, Bihar, India), Dr. Mahendra Pratap Singh (Department of Community Medicine and Family Medicine, All India Institute of Medical Sciences, Jodhpur, Rajasthan, India), and Mr. Arvind Kumar Jaiswal (Department of Community Medicine and Family Medicine, All India Institute of Medical Sciences, Gorakhpur, Uttar Pradesh, India).
